# Prevalence of Sars-Cov-2 Infection in Health Workers (HWs) and Diagnostic Test Performance: The Experience of a Teaching Hospital in Central Italy

**DOI:** 10.3390/ijerph17124417

**Published:** 2020-06-19

**Authors:** Edith Lahner, Emanuele Dilaghi, Claudio Prestigiacomo, Giuliano Alessio, Laura Marcellini, Maurizio Simmaco, Iolanda Santino, Giovanni Battista Orsi, Paolo Anibaldi, Adriano Marcolongo, Bruno Annibale, Christian Napoli

**Affiliations:** 1 Department of Medical Surgical Sciences and Translational Medicine, “Sapienza” University of Rome, Via di Grottarossa 1035/1039, 00189 Rome, Italy; emanuele.dilaghi@uniroma1.it (E.D.); claudio.prestigiacomo@uniroma1.it (C.P.); bruno.annibale@uniroma1.it (B.A.); christian.napoli@uniroma1.it (C.N.); 2 Hospital Direction and Clinical Departments, Sant’Andrea University Hospital, Via di Grottarossa 1035/1039, 00189 Rome, Italy; giualessio1@gmail.com (G.A.); laura.marcellini@ospedalesantandrea.it (L.M.); Maurizio.simmaco@uniroma1.it (M.S.); iolanda.santino@uniroma1.it (I.S.); giovanni.orsi@uniroma1.it (G.B.O.); paolo.anibaldi@ospedalesantandrea.it (P.A.); adriano.marcolongo@ospedalesantandrea.it (A.M.); 3 Department of Neurosciences, Mental Health, and Sensory Organs, “Sapienza” University of Rome, via di Grottarossa 1035-1039, 00189 Rome, Italy; 4 Department of Clinical and Molecular Medicine, “Sapienza” University of Rome, via di Grottarossa 1035-1039, 00189 Rome, Italy; 5 Department of Public Health and Infectious Diseases, “Sapienza” University of Rome, Piazzale Aldo Moro 5, 00185 Rome, Italy

**Keywords:** CoViD-19, health workers (HWs), screening

## Abstract

(1) Background: Health workers (HWs) are at high risk of acquiring SARS-CoV-2 (Severe Acute Respiratory Syndrome Coronavirus 2) infections. Therefore, health authorities further recommend screening strategies for SARS-CoV-2 infection in exposed or high-risk HWs. Nevertheless, to date, the best/optimal method to screen HWs for SARS-CoV-2 infection is still under debate, and data on the prevalence of SARS-CoV-2 infection in HWs are still scarce. The present study aims to assess the SARS-CoV-2 infection rate amongst HWs in a teaching hospital in Central Italy and the diagnostic performance of SARS-CoV-2 serology (index test) in comparison with the SARS-CoV-2 RNA PCR assay (reference standard). (2) Methods: A cross-sectional study on the retrospective data of HWs tested for SARS-CoV-2 by RNA-RT-PCR on nasopharyngeal swabs and by an IgM/IgG serology assay on venous blood samples, irrespective of exposure and/or symptoms, was carried out. (3) Results: A total of 2057 HWs (median age 46, 19–69 years, females 60.2%) were assessed by the RNA RT-PCR assay and 58 (2.7%) tested positive for SARS-CoV-2 infection. Compared with negative HWs, SARS-CoV-2-positives were younger (mean age 41.7 versus 45.2, *p* < 0.01; 50% versus 31% under or equal to 40 years old, *p* < 0.002) and had a shorter duration of employment (64 versus 125 months, *p* = 0.02). Exposure to SARS-CoV-2 was more frequent in positive HWs than in negatives (55.2% versus 27.5%, *p* < 0.0001). In 44.8% of positive HWs, no exposure was traced. None of the positive HWs had a fatal outcome, none of them had acute respiratory distress syndrome, and only one required hospitalization for mild/moderate pneumonia. In 1084 (51.2%) HWs, nasopharyngeal swabs and an IgM/IgG serology assay were performed. With regard to IgM serology, sensitivity was 0% at a specificity of 98.99% (positive predictive value, PPV 0%, negative predictive value, NPV 99.2%). Concerning IgG serology and irrespective of the time interval between nasopharyngeal swab and serology, sensitivity was 50% at a specificity of 99.1% (PPV 28.6%, NPV 99.6%). IgG serology showed a higher diagnostic performance when performed at least two weeks after testing SARS-CoV-2-positive at the RNA RT-PCR assay by a nasopharyngeal swab. (4) Conclusions: Our experience in Central Italy demonstrated a low prevalence of SARS-CoV-2 infection amongst HWs, but higher than in the general population. Nearly half of the positive HWs reported no previous exposure to SARS-CoV-2-infected subjects and were diagnosed thanks to the proactive screening strategy implemented. IgG serology seems useful when performed at least two weeks after an RNA RT-PCR assay. IgM serology does not seem to be a useful test for the diagnosis of active SARS-CoV-2 infection. High awareness of SARS-CoV-2 infection is mandatory for all people, but especially for HWs, irrespective of symptoms, to safeguard their health and that of patients.

## 1. Introduction

Since December 2019, the entire world is still fighting against an infection caused by a novel coronavirus, designated as SARS-CoV-2 (Severe Acute Respiratory Syndrome Coronavirus 2). The epidemic started in China and has spread rapidly worldwide, becoming a pandemic [1]. 

Before the 2009 influenza A(H1N1) pandemic, most European Union (EU) Member States, including Italy, had already developed preparedness plans including planning assumptions on what can be expected during a pandemic and on how a pandemic virus might behave [2], also considering the infective risk related to the global movements of the population [3]. Nevertheless, since the first outbreak of SARS-CoV-2 disease (CoViD-19) at the end of February 2020 in Northern Italy, the epidemic gradually spread across the Country [4]. As of 15 May 2020, Italy has had 223,096 confirmed cases, and 31,368 deaths [1].

From a clinical point of view, the CoViD-19 syndrome is characterized by fever, dry cough, shortness of breath, and in severe cases, by acute respiratory distress syndrome, sepsis, septic shock, and finally multi-organ failure [5]. SARS-CoV-2 is highly contagious and its main route of human-to-human transmission occurs through direct contact or air droplets with a higher risk of transmission within one meter from the infected person [6]. Furthermore, a possible fecal–oral transmission has been described, and feces of SARS-CoV-2-positive patients are potentially infectious [7].

Health workers (HWs), directly or indirectly exposed to actually or potentially positive SARS-CoV-2 patients, may be themselves at an increased risk [8].

Data regarding the prevalence of SARS-CoV-2 infections in HWs are scarce and characterized by underlying limits [9]. The proportion of infected persons is estimated to range between 4.1 and 38.9% and 5.1 and 5.7% among the HWs population and the general population, respectively [9,10,11]. In any case, the overall estimated CoViD-19 incidence, using epidemiologic data for denominators, was higher in HWs than the general population [9]. 

Working in high-risk versus general heath departments, suboptimal handwashing before or after patient contact, longer work hours, and improper protective personal equipment (PPE) use were reported as risk factors for acquiring the disease [9].

From a clinical point of view, in general, HWs appear to experience less severe illness and present lower mortality rates than non-HWs, also related to younger age and fewer comorbid conditions [9]. However, these data are not confirmed in Italy, where an elevated number of fatalities among healthcare personnel was reported [12].

Therefore, many health institutions undertook steps to establish infection prevention measures for creating a safe environment to protect both patients and HWs from SARS-CoV-2 infection. Moreover, international health authorities recommended screening strategies for SARS-CoV-2 infection in exposed or high-risk HWs [13,14,15]; further, during a pandemic, protecting the health of HWs is of paramount importance for reducing morbidity and mortality, reducing transmission, and maintaining the health system capacity [16]. 

As stated by the Italian Ministry for Health, following WHO recommendations, the RNA RT-PCR assay on mucus obtained by nasopharyngeal swabs is considered the reference standard for the diagnosis of SARS-CoV-2 infection [17]. Indeed, the broad use of serology assays for diagnostic purposes is still controversial due to suboptimal diagnostic performance, while these assays may be important for epidemiological purposes [13,14,16,18,19].

Therefore, to date, the best/optimal method to screen HWs for SARS-CoV-2 infection seems still under debate, and data on the prevalence of SARS-CoV-2 infection in HWs working in public hospitals are still scarce. 

In light of this scientific background, the present study aims to assess: (i) the SARS-CoV-2 infection rate amongst HWs in a teaching hospital in Central Italy, and (ii) the diagnostic performance of SARS-CoV-2 serology (index test) in comparison with the SARS-CoV-2 RNA PCR assay (reference standard). 

## 2. Materials and Methods 

### 2.1. Study Population and Design

A cross-sectional study was performed in a teaching hospital in Rome (Central Italy), a secondary referral center with approximately 450 beds and 1,300,000 services provided per year (including those for both inpatients and outpatients). Overall, the hospital accounts for 2057 HWs, distributed in different working categories. During the pandemic, the hospital was indicated as a CoViD-19 regional hub. 

The study was based on retrospective data (18 March–27 April 2020) of HWs (physicians, nurses, other hospital staff). Upon the decision of the general and health management of the hospital, all components of the hospital staff were consecutively tested for SARS-CoV-2 by RT-PCR on nasopharyngeal swabs irrespective of exposure and/or symptoms at suspicion of CoViD-19. Before the nasopharyngeal swabs, the HWs filled in a questionnaire containing demographical data, profession, working unit, and exposure to SARS-CoV-2 infection. From 7 April 2020, the whole staff were also invited to be tested for the presence of IgM/IgG antibodies against SARS-CoV-2 using serology assays on venous blood samples on a voluntary basis, and a substantial subset (50.2%) adhered to this initiative. 

HWs were considered positive to SARS-CoV-2 infection when they tested positive to the RT-PCR (reference standard). SARS-CoV-2-positive and -negative HWs were compared for clinical data and exposure to SARS-CoV-2 infection. In the subset of HWs tested by both RT-PCR and serology, results of the IgM and IgG serology assays were compared to RT-PCR (index test) to assess the diagnostic performance of the serology tests.

This paper was drafted according to STROBE and STARD guidelines to ensure the quality of reporting [20,21].

### 2.2. Laboratory Tests of SARS-CoV-2 Infection

SARS-CoV-2 RT-PCR was performed on mucus obtained from nasopharyngeal swabs by a commercial kit according to the manufacturer’s instructions (Allplex™ 2019-nCoV Assay, Seegene Inc., Seoul, Korea). 

IgM and IgG SARS-CoV-2 serology was performed on venous blood samples by a commercial chemiluminesce immunoassay (CLIA), (Medical Systems, 2019-nCoV IgM/IgG, Genova, Italy) according to the manufacturer’s instructions.

### 2.3. Statistical Analyses

Descriptive statistics were expressed by number (percentage) of total, mean ± SD, and median (range).

Differences between RT-PCR-positives and -negatives were analyzed by chi-square and Fisher’s tests as appropriate for the categorical variables and by a Student’s *t*-test or Mann–Whitney-test for continuous variables. Diagnostic performance of the serology assay (index test) was computed in comparison with the RT-PCR assay and expressed as sensitivity, specificity, positive (PPV), and negative predictive values (NPV). 

Statistical analyses were performed by MedCalc© Statistical Software version 19.0.4 (MedCalc Software bvba, Ostend, Belgium).

### 2.4. Ethical Issue

The study was performed following the World Medical Association Declaration of Helsinki and did not include any identifiable human data. Approval of Sapienza University of Rome Ethical Committee was obtained (N. 7010/2020).

## 3. Results

A total of 2057 HWs (median age 46, 19–69 years, females 60.2%) were assessed by the RT-PCR assay and 58 (2.7%) tested positive for SARS-CoV-2 infection. As detailed in Table 1, SARS-CoV-2-positive HWs were younger (mean age 41.7 versus 45.2 years, *p* < 0.01; 50% versus 31% under or equal to 40 years old, *p* < 0.002) and had a shorter duration of employment (64 versus 125 months, *p* = 0.02) than negative HWs. Exposure to SARS-CoV-2 was more frequent in positive HWs than in negatives (55.2% versus 27.5%, *p* < 0.0001). In 44.8% of positive HWs, no exposure was traced. No differences between SARS-CoV-2-positives and -negatives were observed concerning gender and profession. 

Taking into consideration only those HWs who had previous exposure to infection, only the CoViD-19 wards had a significantly higher proportion of SARS-CoV-2-positives than -negatives (18.7% versus 5.7%, *p* = 0.0035), while in the other work areas, positives and negatives were equally distributed. 

Amongst the SARS-CoV-2-positive HWs, 67.3% had associated symptoms, most frequently fever (34.7%), ageusia (34.7%), anosmia (26.5%), cough (22.4%), asthenia (20.4%), and arthralgia/myalgia (20.4%). None of the positive HWs had a fatal outcome, none of them had acute respiratory distress syndrome, and only one required hospitalization for mild/moderate pneumonia (Table 2). 

### Diagnostic Performance of SARS-CoV-2 Serology

The overall seroprevalence in our study population was 0% for IgM and 0.7% for IgG antibodies. 

In 1084 (51.2%) HWs (aged ≤ 40 years 30.4%, females 60.9%), both nasopharyngeal swabs for the RT-PCR and IgM/IgG serology assays were performed. The main characteristics of this subset of HWs did not differ from those of the whole study population. The median interval between the swabs and serology assays was 10 (0–34 days). 

**Figure 1:**
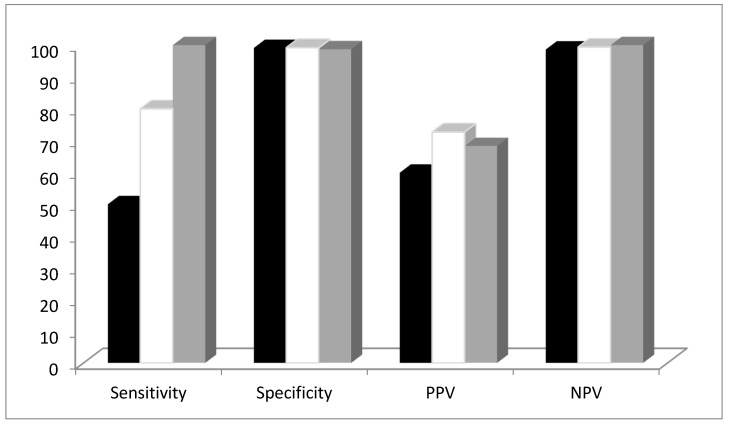
Diagnostic performance of SARS-CoV-2 IgG serology (index test) in comparison with RT-PCR (reference standard) considering a time interval of at least 14 days and 20 days between swabs and serology. PPV = positive predictive values, NVP = negative predictive value. 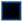 Irrespective of the time interval between serology and positivity to RT-PCR. 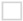 14 days after positivity to RT-PCR. 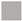 20 days after positivity to RT-PCR.

As shown in Table 3A, concerning IgM serology, sensitivity was 0% at a specificity of 98.99% (PPV 0%, NPV 99.2%); concerning IgG serology and irrespective of the time interval between the nasopharyngeal swab and serology, sensitivity was 50% at a specificity of 99.1% (PPV 28.6%, NPV 99.6%). The diagnostic performance of IgG serology substantially improved considering only the serology assays performed at least 14 days (sensitivity 80%, specificity 99.2%, PPV 50%, NPV 99.8%) or 20 days (sensitivity 100%, specificity 98.7%, PPV 57.1%, NPV 100%) after the nasopharyngeal swab (Table 3B, Figure 1). 

## 4. Discussion

The present study evaluated the SARS-CoV-2 infection prevalence amongst HWs in an academic hospital in Latium, a low-incidence region located in Central Italy. The Latium region has 5,890,401 inhabitants [22] and by 24 April 2020, a total of 4492 SARS-CoV-2-positive cases was registered [23], corresponding to 2.3% of all positive cases in Italy. Based on these data, an overall estimate of SARS-CoV-2 infection prevalence of 0.08% in the general Latium population can be calculated [22,23]. The 2.3% prevalence of SARS-CoV-2 infection amongst the HWs in our teaching hospital would thus correspond to a 34 times higher occurrence of positivity (2.7%). This prevalence amongst HWs reflects the expected higher job-related-risk of infection [8]. Further, due to the virus’s characteristics of contagiousness and route of transmission, HWs, providing for medical and sanitary care of SARS-CoV-2-infected patients, are directly or indirectly exposed to infection, and are themselves at higher risk of being infected, notwithstanding accurate hygiene and personal protective devices [16]. Nevertheless, compared with a national rate of 10.7% of SARS-infected HWs [12], in our teaching hospital, identified as one of the CoViD reference facilities in the Latium region, the rate of infected HWs was more than three times lower, thus suggesting an effective and timely strategy to limit the spread of SARS-CoV infection. Indeed, according to the guidelines provided by the National Institute of Health [24], in our hospital, prevention protocols were timely implemented: strict hygiene rule for hand washing before and after all patients interactions, contact with potentially infectious sources, and before putting on and after removal of personal protective equipment; wearing of personal protective equipment for all HWs (typically composed by surgical face mask, and white cotton gown or white or green dresses, hairnet, goggles, gloves, surgical mask, and disposable gown, in the case of low-risk patients, and hairnet, googles or face-shield, FFP2-3 mask, water-resistant gown with long sleeves, and two pairs of gloves (second one covering the wrist of gown sleeves) in the case of high-risk or nasopharyngeal swabs positive patients); measurement of body temperature at the hospital entry (subjects with body temperature over 37.5 °C were not allowed to enter in the building); different ways for clean and dirty material; separate doors for entry and exit; accurate patients’ hand hygiene, etc. These data are further confirmed by the fact that only 29 (50%) of the 58 SARS-CoV-2-infected HWs declared to have had an exposition to infected patients inside the hospital, thus probably further reducing the rate of hospital-related infections. The effectiveness of prevention measures in reducing the spread of infection to HWs has been demonstrated in other studies that reported a zero rate of HWs being infected also after performing high-risk procedures [25].

In the vast majority, SARS-CoV-2-positive HWs had a mild course of infection (67%), the remaining percentage showed no symptoms at all; in any case, none had a fatal outcome or severe pneumonia. In the non-symptomatic HWs, SARS-CoV-2 infection was diagnosed only thanks to the proactive screening strategy performed in all HWs. This infection prevention strategy might have further contributed to limit the spread of infection amongst HWs, but also amongst patients, leading to a timely diagnosis and subsequent safety measures in these HWs unaware of being infected. Paucisymptomatic or symptomless carriers of infection play a crucial role in spreading the infection. Further, in the UK, a screening program on asymptomatic HWs in a teaching hospital swabbed and tested by RT-PCR was carried out, and 3% tested positive for SARS-CoV-2 infection [26], thus keeping in step with our results. On the other hand, a study on SARS-CoV-2 testing in HWs found a symptoms-related performance of diagnosis by RT-PCR [27]; therefore, great attention must be paid to which symptoms should be taken into account for building the screening decisional algorithm. Taken together, these data suggest that proactive molecular screening strategies including asymptomatic as well as symptomatic HWs should be a priority at the national and international levels. 

The all-testing strategy in our healthcare facility likely had another indirect positive implication on HWs concerning psychological distress during the pandemic [28], which is increasing and at risk of leading to HW burnout [29]. Further, if not specifically assessed in this study, getting tested and thus obtaining certainty about the proper infection status might contribute to reducing the psychological burden and fear of being infected of HWs during the pandemic.

In our study, the SARS-CoV-2-positive HWs were younger than the negatives with half of them being younger than 40 years of age. This result is not consistent with other studies that found no association between age and SARS-CoV-1 [9], while concerning SARS-CoV-2, a very recent Chinese study reported a 1.9 higher occurrence of SARS-CoV-positivity in HWs younger than 45 years of age compared with over 45 years, thus supporting our finding [30]. Our results might be explained by less work experience due to the younger age, as also supported by the shorter duration of employment of positive compared with negative HWs. 

Concerning SARS-CoV serology, the overall IgG seroprevalence was low (0.7%), when compared with the infection rate detected by the molecular RT-PCR assay (2.7%). A German study, performed in a tertiary referral hospital, described in HWs, stratified for confirmed, suspected, or no infection exposure, a seroprevalence of 1.6%, but the staff were not systematically tested by RT-PCR [31]. Moreover, our study further showed that the diagnostic performance of SARS-CoV-IgG serology is time-dependent, sensibly increasing when performed after at least two weeks from an RT-PCR documented positivity. Sensitivity increased from 50%, when calculated irrespective of the time interval between RT-PCR and serology, to 80% after 14 days, and to 100% after 20 days of RT-PRC. These data seem consistent with the results obtained in another study performed with a chemiluminescent assay different from that used in our study. It showed an 88.7% sensitivity at 7 days and a 100% sensitivity at 14 and 17 days after PCR positivity with specificity values of 99% [32]. 

One further study using a magnetic chemiluminescence enzyme immunoassay (MCLIA) for virus-specific antibody detection showed that, within 19 days after the onset of symptoms, 100% of patients tested positive for IgG anti-SARS-CoV-2, supporting our results. However, in this study, seroconversion for IgG and IgM occurred almost simultaneously, and approximately 20–22 days after symptom onset, 94% of patients showed positivity to virus-specific IgM antibodies [33]. On the contrary, in our experience, the diagnostic performance of IgM serology was lower than IgG, showing 0% of sensitivity at 99% specificity as none of the RT-PCR-positive subjects showed an IgM seroconversion.

These data, taken together, confirm the likely ideal window of two weeks for the broad seroconversion and clear detectability of anti-SARS-CoV-2 IgG antibodies, while factors influencing the seroconversion of IgM antibodies are less clear, with IgM detectability being also assay-dependent [19]. 

Therefore, IgM serology does not always appear useful for diagnostic purposes of active SARS-CoV-2 infection, while IgG serology has been suggested to be used for three purposes in the SARS-CoV-2 pandemic: (1) to diagnose infection in a limited niche of patients, (2) to identify convalescent plasma donors, and (3) to screen populations with the purpose to determine exposure and immunity [34]. However, after much clamor generated around serologic assays, there is still a need for data to support their clinical utility to avoid to misdiagnose and misinform [34]. 

Finally, it should be noted that the available serologic assays are very different in their format (enzyme-linked immunosorbent assays, chemiluminescent immunoassays, lateral flow immunoassays, virus neutralization assays), in the detected antibody class (IgA, IgM, IgG, or pools), in the used SARS-CoV-2 antigens (for example the recombinant nucleocapsid protein, the subunit 1 of the spike glycoprotein, or the spike glycoprotein receptor binding domain), and in the specimen on which the assay is performed (plasma, serum, whole blood, finger stick) [35,36,37,38]. High-quality serological assays are now becoming available, which need to be strategically applied, deployed, and validated to implement their use for diagnostic, therapeutical, and epidemiological purposes.

We are aware of some limits of the study. This was a single-center study conducted in Central Italy, probably not able to reflect the spread of infection in HWs throughout the whole country. To minimize the effects of this limit, for the comparison of the infection rate of HWs with the general population, only the estimated data of the Latium region in the middle of April were taken into consideration. Moreover, due to organizational reasons, the nasopharyngeal swabs for SARS-CoV-2 infection were performed over a time frame of 40 days. Therefore, we are not able to exclude that HWs who tested negative at the beginning of the study might have become positive over time. However, during this period, the other prevention measures, such as body temperature measurement, would probably have allowed ascertaining these cases; however, we did not analyze the extent to which the control measures were accomplished. Further, only half of the HWs joined the serology screening for the detection of anti-SARS-CoV-2 antibodies, thus reducing the sample number for the statistical computations.

## 5. Conclusions

Our experience in Central Italy demonstrated a low prevalence of SARS-CoV-2 infection amongst HWs, but higher than in the general population. Nearly half of the positive HWs reported no previous exposure to SARS-CoV-2-infected subjects and were diagnosed thanks to the proactive screening strategy put in place. IgG serology showed a higher diagnostic performance when performed at least two weeks after testing SARS-CoV-2-positive at the RNA RT-PCR assay by a nasopharyngeal swab. IgM serology seems not to be a useful test for the diagnosis of active SARS-CoV-2 infection. High awareness of SARS-CoV-2 infection is mandatory for all people, but especially for HWs, irrespective of symptoms, to safeguard their health and that of patients. 

## Figures and Tables

**Table 1:** Comparison between main characteristics of the RT-PCR SARS-CoV-2-positive and -negative health care workers (HWs).

	SARS-CoV-2-Positive HWs *n* = 58 (2.7%)	SARS-CoV-2-Negative HWs *n* = 2057 (97.3%)	*p*
Age, years, mean ± SD	41.7 ± 10.5	45.2 ± 11.1	0.0187
Age ≤ 40 years	29 (50)	637 (31)	0.0021
Duration of employment, months, median (range)	64 (1–229)	125 (0–232)	0.0176
Females	37 (63.8)	1238 (60.2)	0.5798
Health workers	53 (91.4)	1284 (62.4)	0.0016
- Physicians	26 (44.8)	606 (29.5)	0.0979
- Nurses	27 (46.5)	678 (32.9)	0.2093
- Other hospital staff	5 (8.6)	773 (37.6)	0.0016
Exposure to SARS-CoV-2-positive subjects	32 (55.2)	565 (27.5)	<0.0001
- Inhouse exposure	29 (50.0)	565 (27.5)	0.0002
- Outside exposure	3 (5.2)	0 (0.0)	<0.0001

Data are expressed as number (%) when not otherwise indicated.

**Table 2:** Characteristics of SARS-CoV-2-positive health care workers.

Characteristics	Data Title
Females	63.8
Age, years, median (range)	40.5 (24–65)
Age ≤ 40 years	50
Body mass index, kg/m^2^, median (range)	24.3 (18.7–31.2)
Smoking	25
Regular sport/fitness	35.7
Presence of comorbidities	30.2
New employees (employed less than 1 month)	8.6
CoViD-19 characteristics	
Presence of symptoms	67.3
- Fever	34.7
- Ageusia	32.6
- Anosmia	26.5
- Cough	22.4
- Asthenia	20.4
- Arthralgia/myalgia	20.4
- Diarrhea	14.3
- Dyspnea	10.2
- Conjuntivitis	8.2
- Headache	8.2
- Other symptoms (rhinorrhea, dizziness, chill, rash)	28
Thorax CT confirmed Interstitial pneumonia	16.7
ARDS	0.0
Hospitalization	1.7

Data are expressed as %.

**Table 3:** (**A**) Diagnostic performance of SARS-CoV-2 IgM and IgG serology (index test) in comparison with RT-PCR (reference standard) irrespective of the time interval between swabs and serology. (**B**) Diagnostic performance of SARS-CoV-2 IgG serology (index test) in comparison with RT-PCR (reference standard) considering a time interval of at least 14 days and 20 days between swabs and serology.

**(A)**		
**IgM Serology Assay**	**Rate**	**95% Confidence Interval**
Sensitivity	0.00%	0.0% to 36.9%
Specifity	98.90%	98.2% to 99.5%
AUC	0.5	0.5 to 0.5
Positive Likelihood Ratio	0	
Negative Likelihood Ratio	1	1.0 to 1.0
Positive Predictive Value	0.00%	
Negative Predictive Value	97.30%	97.2% to 97.3%
Accuracy	96.30%	95.0% to 97.3%
**IgG Serology Assay**	**Rate**	**95% Confidence Interval**
Sensitivity	50.00%	15.7% to 84.3%
Specificity	99.10%	98.3% to 99.5%
AUC	0.7	0.7 to 0.8
Positive Likelihood Ratio	53.8	21.3 to 136.0
Negative Likelihood Ratio	0.5	0.2 to 1.0
Positive Predictive Value	59.90%	37.1% to 79.1%
Negative Predictive Value	98.60%	97.3% to 99.3%
Accuracy	97.70%	96.7% to 98.5%
**(B)**		
**IgG Serology After 14 Days**	**Rate**	**95% Confidence Interval**
Sensitivity	80.00%	28.3% to 99.5%
Specificity	99.20%	97.9% to 99.8%
AUC	0.9	0.9 to 0.9
Positive Likelihood Ratio	96	32.9 to 279.8
Negative Likelihood Ratio	0.2	0.0 to 1.2
Positive Predictive Value	72.70%	47.7% to 88.6%
Negative Predictive Value	99.40%	96.9% to 99.9%
Accuracy	98.60%	97.2% to 99.5%
**IgG Serology After 20 Days**	**Rate**	**95% Confidence Interval**
Sensitivity	100.00%	39.8% to 100.0%
Specificity	98.70%	96.3% to 99.7%
AUC	0.9	0.9 to 1.0
Positive Likelihood Ratio	77.7	25.2 to 239.1
Negative Likelihood Ratio	0	
Positive Predictive Value	68.30%	41.2% to 86.9%
Negative Predictive Value	100.00%	
Accuracy	98.70%	96.4% to 99.7%
